# Trends and patterns of inequality in modern contraceptive use in urban and rural India: are family planning programmes increasingly reaching the marginalized?

**DOI:** 10.1093/heapol/czaf073

**Published:** 2025-11-12

**Authors:** Abhishek Kumar, Subrato Kumar Mondal, Ashita Munjral, Rajib Acharya, Niranjan Saggurti

**Affiliations:** PopulationCouncil Consulting Pvt. Ltd., Zone-5A, Ground Floor, India Habitat Centre, Lodi Road, New Delhi 110003, India; USAID/India, American Embassy, Chanakya Puri, New Delhi 110021, India; Population Council, India Country Office, New Delhi 110003, India; Population Council, India Country Office, New Delhi 110003, India; Population Council, India Country Office, New Delhi 110003, India

**Keywords:** family planning, inequality, concentration index, NFHS, India

## Abstract

India has made good progress in the use of modern contraceptives in recent decades, however identifying women who are left behind is important to policy makers for further improving availability, accessibility, and coverage of family planning services to the marginalized population and hence achieving the international and national development agenda. Using five rounds of the National Family Health Survey data conducted between 1992–93 to 2019–21, this study examined the trends and patterns in inequality—by household wealth quintile and women’s education—in modern contraceptive prevalence rates (mCPR) and demand for family planning satisfied with modern methods in urban and rural areas. The findings showed a secular trend of increasing rates in the use of modern contraceptives across socioeconomic sub-groups within urban (mCPR among the poorest quintile increased from 32% to 49%, and among the richest quintile from 51% to 60% in 1992–93 to 2019–21, respectively) and rural (mCPR among the poorest quintile increased from 27% to 49%, and among the richest quintile from 49% to 59% in 1992–93 to 2019–21, respectively) areas. Similarly, the inequality over time—measured by the concentration index—in mCPR has declined from 0.311 to 0.158 in urban areas and from 0.247 to 0.143 in rural areas between 1992–93 to 2019–21. Despite the overall decline in inequality, the pro-rich situation persists in contraceptive use in the country, and the extent of the inequality was high for modern reversible methods, both in urban and rural areas. Our findings underscore the increasing availability and accessibility of modern reversible methods, particularly among marginalized populations, along with improved information provided on the range of choices. This will help in achieving the global commitment of universal access to reproductive health, including family planning, and balance the method-mix in a country that is currently dominated by female sterilization.

Key messages

- In India, overall and within urban–rural areas, a substantial increase in modern contraceptive use among the poorest and uneducated women indicates that family planning programmes have been increasingly reaching the marginalized.
- The relative increase in modern contraceptive use among poor and uneducated women (compared to their counterparts) might be contributed to by decreasing inequality over time, within both urban and rural areas.
- Despite decreasing inequality, the level of inequality is persistent (reflecting the pro-rich situation) and comparatively higher for modern reversible methods of contraception than for other indicators. This may be because affluent women are more likely to procure the reversible contraceptives from the private sector at their own expense, which marginalized women may not be able to do.
- Relative change in modern contraceptive uptake is higher among marginalized rural women than among their urban counterparts, which may be the result of community-based programmes of demand generation by frontline health workers, which has been more intensive within rural areas.

## Introduction

In India, the family planning (FP) programme has made impressive progress in recent years—>50% of married women started using modern contraceptive methods and the majority of them opted for their method based on informed choice, demand for FP satisfied with modern methods is close to the FP2020 target, and the total fertility rate dwindled below the replacement level of fertility (IIPS and ICF 2021). Going forward, the country has committed to extend and strengthen its FP programme as FP2030 by expanding the access and availability of FP information and services to meet the FP needs of women who are in need, with a focus on those who are marginalized. While continued efforts and achievement in the FP programme is commendable, it is also important to examine the progress with an equity lens to ensure that regardless of socioeconomic status, all women and couples can use the method that meets their needs (FP2030). This is even more important as India is signatory to one of the Sustainable Development Goals (SDG 3.7) which committed to provide access to voluntary and quality FP services to ensure universal access to sexual and reproductive healthcare, including FP aimed at ‘leaving no one behind’ (Raj and McDougal 2017). The present paper, therefore, examines trends and patterns of inequality in FP programme coverage in India across urban and rural areas.

Although contraceptive prevalence rates have increased globally, pro-rich socioeconomic inequality continuously persists (Gakidou and Vayena 2007, Creanga et al. 2011, Aslam et al. 2016, Ugaz et al. 2016, Gonçalves et al. 2019, Hellwig et al. 2019, Ponce de Leon et al. 2019, Mutua et al. 2021, Das et al. 2022, Sharma and Singh 2022, Sreeramareddy et al. 2022). Women with low socioeconomic status are outpaced by well-off women in contraceptive use due to several reasons, ranging from low knowledge about contraceptive methods available and related side-effects (Sedgh and Hussain 2014, Rios-Zertuche et al. 2017, Moreira et al. 2019), limited accessibility to FP services (Prata 2009, Bongaarts 2014), low affordability when method of choice is not available free (Bailey et al. 2019), propensity of high fertility desire (Paudel and Acharya 2018), poor maternal and child health including high mortality rates (Prata et al. 2009, UNICEF 2009), inability to overcome barriers such as prevailing gender and social norms, and familial and religious prohibition that prevents women from accessing and using contraceptives (Kragelund Nielsen et al. 2012, Mboane and Bhatta 2015, Machiyama et al. 2017, de Vargas Nunes Coll et al. 2019, Moreira et al. 2019). These factors suppress contraceptive use among poor and less-educated women compared with their richer and better-educated counterparts.

The inequality in contraceptive use differs by type of method and region as well due to different policy environments. For instance, in countries like India and Bangladesh, where contraceptive use is associated with supply- and demand-side incentives, very low inequality is found in the use of long-acting permanent methods, whereas considerable pro-rich inequality persists in the use of short-acting contraceptive methods (Ugaz et al. 2016). On the other hand, in African and Latin American countries, where unintended births are high, spousal support in FP is low, and health service providers lack the knowledge and skill to provide permanent methods, poorer women rely heavily on short-acting reversible contraceptive methods whereas richer women are likely to use long-acting reversible or permanent methods (Fotso et al. 2013, Ugaz et al. 2016).

In the current paper, we intend to examine trends and patterns of socioeconomic inequality—by household wealth quintiles and education level among women—in contraceptive prevalence rate by modern methods, modern non-reversible methods (MNRMs), modern reversible methods (MRMs), and demand for FP satisfied with modern contraceptive methods. Identifying women who are left behind is important for ongoing FP programmes to improve the availability, accessibility, and reach of FP services for them. This will help in achieving the international and national development agenda related to FP. To achieve the 75% target of demand for FP satisfied with modern methods, India needs ∼22.5 million additional users of modern contraceptives by 2030 (New et al. 2017). Meeting the contraceptive needs of such a huge number of women is important for achieving universal access to sexual and reproductive health for all (SDG 3.7), and to ensure the reproductive rights of women (SDG 5.6) (Blumenberg et al. 2020).

Although previous studies in India documented economic inequality in FP, the analyses are restricted to only one time-point data, a specific group of the population, limited indicators, and specific geography (Kumar et al. 2020b, Ewerling et al. 2021, Das et al. 2022, Sharma and Singh 2022, Sreeramareddy et al. 2022). Moreover, these studies focused on overall inequality and did not account for differences in urban and rural areas. Currently, rapid urban growth in developing countries including India is taking place in smaller cities that are not equipped institutionally to deal with the challenges arising from this rapid natural increase (United Nations 2019). Furthermore, where poverty is likely to be concentrated in urban rather than rural areas and where urban inequalities are deepening, merely comparing urban–rural differences in contraceptive use is insufficient (Vearey et al. 2019). Hence, understanding the trends and patterns of inequality in FP within urban and rural areas will help with the adoption of different programmatic strategies.

## Methods

### Data

This study analysed cross-sectional data of all five rounds of the National Family Health Survey (NFHS) of India, conducted during 1992–93, 1998–99, 2005–06, 2015–16, and 2019–21. The NFHS of India collects data in a similar format to the Demographic Health Survey (DHS) in other countries. All rounds of the NFHS are nationally representative surveys of households and women of reproductive age (15–49 years), which were selected by adopting a multi-stage stratified sampling design. All surveys were stratified by urban–rural residence and by the states of the country. The NFHS 2015–16 and 2019–21 was stratified at the sub-state level as well and provides estimates on FP and maternal and child health indicators at the district level. The women’s response rate was high in all rounds of the NFHS—96% each in 1992–93 and 1998–99, 95% in 2005–06, and 97% each in 2015–16 and 2019–21. The analysis of this paper is based on samples of currently married women (aged 15–49 years) of 84 328, 84 682, 93 088, 499 627, and 512408 in the NFHS 1992–93, NFHS 1998–99, NFHS 2005–06, NFHS 2015–16, and NFHS 2019–21, respectively.

### Measures

#### Outcome variables

In this study, four FP indicators were considered as key outcome variables. Those outcome variables are defined as follows. Modern contraceptive prevalence rate (mCPR): mCPR is the standard outcome indicator to track the progress of FP planning programmes across the globe. mCPR is defined as the percentage of currently married women aged 15–49 years who were using any modern method of contraceptive at the time of the survey. In all rounds of the NFHS, the currently married women were asked ‘Are you currently doing something or using any method to delay or avoid getting pregnant?’ Those women who responded ‘yes’ were further asked ‘Which method are you using?’ Women who responded that they/their husbands were using either of female sterilization, male sterilization, an intrauterine contraceptive device, male/female condoms, oral contraceptive pills, injectables, diaphragm, and the standard days method etc. were considered as users of modern contraceptives. All the currently married women aged 15–49 years who adopted female sterilization or their husbands adopted male sterilization were considered as using a MNRM. All the currently married women aged 15–49 years who were using either an intrauterine contraceptive device, male/female condoms, oral contraceptive pills, injectables, diaphragm and the standard days method etc. at the time of survey were considered as users of MRMs.

Demand for FP satisfied with a modern method (demand satisfied) is defined as: among currently married women aged 15–49 years, the proportion of women’s demand for FP that is satisfied with modern contraceptives. This is a composite indicator computed using current use of any contraceptive, current use of any modern contraceptive, and unmet need for FP.

Based on these three indicators, met demand is defined as follows:


$$Demand\;satisfied=\frac{\mathrm{current}\;\mathrm{contraceptive}\;\mathrm{use}\;(\mathrm{modern}\;\mathrm{method})}{\mathrm{unmet}\;\mathrm{need}+\mathrm{current}\;\mathrm{contraceptive}\;\mathrm{use}\;(\mathrm{any}\;\mathrm{method})}$$

In the following, ‘demand for FP satisfied with a modern method’ is interchangeably used with ‘demand satisfied’.

#### Predictor variables

In this study, household wealth quintiles and women’s education are key predictors considered to capture the socioeconomic status of women and analysed for examining inequality in the selected outcome variables. Many previous studies have used these two variables while examining inequality in contraceptive use (Creanga et al. 2011, Fotso et al. 2013, Ponce de Leon et al. 2019, Sreeramareddy et al. 2022).

In the third, fourth, and fifth rounds of the NFHS, a wealth index was computed—using the variables of household amenities, consumer durables, household goods and assets, size of land holding etc.—by applying principal component analysis, and the index was divided into five wealth quintiles. However, in the first two rounds of the NFHS, a standard of living index was computed based on the arbitrary scoring of economic proxies and the index was divided into three categories. Additionally, the number of variables used in computing the standard of living index or wealth index changed in the subsequent rounds of the NFHS. Therefore, in this study, to make the wealth index comparable over the survey rounds in terms of number of variables and the index categories, a separate wealth index was computed using a common set of variables available in all five rounds of the NFHS by adopting the standard methodology (Montgomery et al. 2000, Filmer and Pritchett 2001, Rutstein and Johnson 2004, Vyas and Kumaranayake 2006, Gwatkin et al. 2007, O’Donnell et al. 2008). The index was divided into five quintiles (20% each): poorest, poorer, middle, richer, and richest. Women’s level of education is computed using the information on number of years of schooling and divided into four categories—no/0 years of schooling, 1–10 years of schooling, 11–12 years of schooling, 12+ years of schooling. For convenience, these categories are named as no education, 1–10 years, 11–12 years, 12+ years, respectively.

Several contextual variables such as age of women, number of living children, caste and religion of women, exposure to FP messages through media, and region of the country are adjusted in the analysis. These variables are found to be associated with adoption of FP services and have been used in previous studies (Creanga et al. 2011).

### Analysis

Bivariate analysis was carried out to examine the prevalence of outcome variables by household wealth quintiles and completed years of schooling across urban–rural residence over the survey period. Inequality was measured by computing absolute difference between the best and the worst group and by taking the ratio of the best to the worst group within the socioeconomic indicator. Furthermore, the concentration index was computed to measure overall socioeconomic inequality in FP indicators by urban–rural residence. The concentration index was defined with reference to the concentration curve, which plots cumulative percentage of outcome variable on the *y*-axis against cumulative percentage of currently married women ranked by household wealth, starting from the poorest and ending with the richest quintile, on the *x*-axis. In an ideal situation, when all the women, irrespective of their economic status ‘*x*’, have the same ‘*y*’, the concentration curve follows a 45-degree line (line of equality), running from the bottom left-hand corner to the top right-hand corner. If ‘*y*’ takes higher values among poorer people, the concentration curve lies above the line of equality. The opposite is true if ‘*y*’ takes a lower value. The greater the distance of the curve from the line of equality, the greater is the economic inequality in ‘*y*’ (O’Donnell et al. 2008). The concentration index is a measure of this inequality and is defined as twice the area between the concentration curve and line of equality (Wagstaff and Doorslaer 2004).

The value of the concentration index varies between −1 and +1, where a negative value indicates that the concentration curve is above the line of equality (the outcome variable is concentrated among poor population) and a positive value indicates that the curve is below the line (the outcome variable is concentrated among rich population), and zero value indicates no inequality. We used the factor score of household wealth, obtained from the principal component analysis, to estimate the concentration index across household economic status. The concentration index for women’s education is based on completed years of schooling (Sastry 2004).

In this study, all the four outcome variables were dichotomous (1 = using contraceptive; 0 = otherwise), hence we applied binary logistic regression analyses in all five rounds of the survey, separately for urban and rural areas. Regression analysis was applied to examine the association between the outcomes and the household wealth quintiles and education among women after adjusting for the selected covariates. After obtaining odds ratios from the regression analysis, a post-estimation command was applied to obtain predicted probabilities (based on the odds ratios) from the model. Hence, we preferably presented the multivariate results in terms of predicted probabilities (along with the 95% confidence interval) instead of odds ratios, for easier interpretation. Given that the NFHS used a multistage sampling design, standard errors were adjusted for weighting and clustering in all estimations by applying the appropriate weight. The analyses presented in the subsequent sections were carried out in STATA 16.0.

## Results

### Trends in modern contraceptive use by urban–rural residence in India

**Figure 1.**
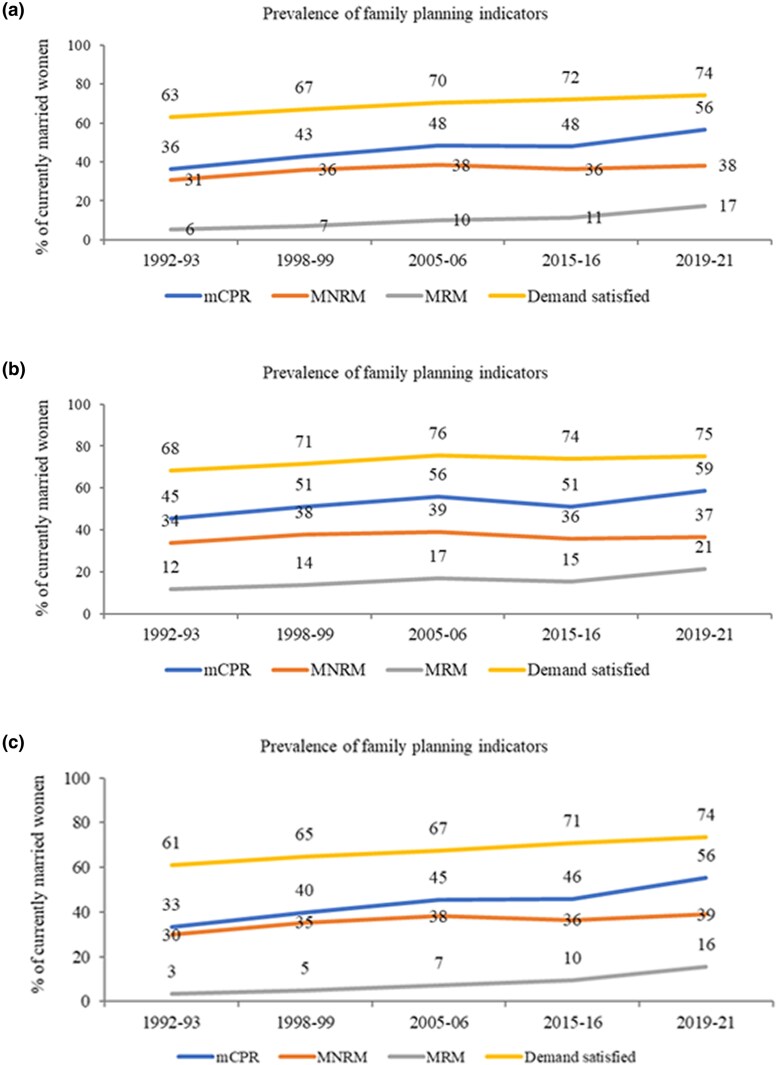
Trends in coverage of FP indicators among currently married women aged 15–49 years in (**a**) India, 1992–2021, (**b**) urban India, 1992–2021, and (**c**) rural India, 1992–2021.

The prevalence of modern contraceptive use and demand satisfied increased in India during 1992–2021—mCPR increased from 36% to 56%, MRM increased from 6% to 17%, and demand satisfied increased from 63% to 74% during the period (Fig. 1a). This trend was similar for urban and rural areas. For instance, in urban areas, mCPR increased from 45% to 59% and MRM increased from 12% to 21% from 1992–93 to 2019–21, respectively (Fig. 1b). Similarly in rural areas, mCPR increased from 33% to 56%, MNRM increased from 30% to 39%, and MRM increased from 3% to 16% during the period (Fig. 1c).

### Trends and differentials in modern contraceptive use by household wealth and education

Over time, contraceptive use and demand satisfied increased across five wealth quintiles, both within urban and rural areas (Table 1). For instance, mCPR increased from 27% to 49% among the poorest women, 30% to 55% among poorer women, 33% to 58% among middle women, 41% to 59% among richer women, and 50% to 60% among the richest women. A similar trend was observed within urban and rural areas. In urban areas, MRM increased from 2% to 16% among the poorest, 2% to 19% among poorer, 4% to 19% among middle, 6% to 20% among richer, and 17% to 24% among the richest quintile from 1992–93 to 2019–21. Despite this increase, there was a gradient in contraceptive use across the five wealth quintiles—lower to higher use among poorest to richest women. For instance, in 2019–21 mCPR was 49% among women from the poorest quintile, 55% among the poorer quintile, 58% among the middle quintile, 59% among the richer quintile, and 60% among the richest quintile. A similar gradient was observed for MRM and demand satisfied but not for MNRM. Contraceptive use also increased across level of education among women (Table 2). Between 1992–93 and 2019–21, mCPR increased from 31% to 60% among women with no education, 45% to 58% among women with 1–10 years of schooling, and 43% to 50% among women with 11–12 years of schooling. This trend was similar within urban and rural areas.

**Table 1.** Trends in coverage of FP indicators among currently married women aged 15–49 years by household wealth quintiles across place of residence in India, 1992–2021.

	Total					Urban					Rural				
	1992–93	1998–99	2005–06	2015–16	2019–21	1992–93	1998–99	2005–06	2015–16	2019–21	1992–93	1998–99	2005–06	2015–16	2019–21
**mCPR**															
Poorest	27.4	28.2	31.9	34.5	49.1	31.8	37.3	32.2	36.5	48.5	27.2	28.0	31.9	34.5	49.1
Poorer	29.7	35.9	44.4	47.5	55.4	32.0	37.1	44.4	46.2	54.4	29.5	35.9	44.4	47.7	55.6
Middle	33.4	44.7	53.0	51.3	58.3	35.3	43.1	51.1	49.2	56.6	33.2	45.0	53.5	52.0	59.0
Richer	40.5	50.0	56.8	51.9	59.4	41.3	48.9	56.6	50.5	58.5	40.2	50.6	56.9	53.4	60.1
Richest	50.1	55.2	58.6	54.0	60.1	50.5	55.2	58.6	53.8	60.4	48.9	55.1	58.8	54.7	59.2
**MNRM**															
Poorest	26.2	26.1	27.7	27.8	32.8	29.6	33.6	27.6	27.6	30.6	26.0	26.0	27.7	27.8	32.9
Poorer	27.9	33.1	38.3	37.0	37.9	30.4	33.6	37.6	34.3	34.1	27.7	33.0	38.4	37.3	38.4
Middle	30.4	40.4	45.7	40.3	41.7	31.6	38.3	43.4	35.7	37.4	30.2	40.8	46.3	41.9	43.2
Richer	34.9	42.4	44.1	39.3	41.3	35.0	40.3	43.0	37.1	37.8	34.8	43.6	45.2	41.7	44.2
Richest	34.7	38.1	36.9	37.1	37.5	34.0	37.0	35.6	36.0	36.2	36.8	41.1	41.7	41.0	40.7
**MRM**															
Poorest	1.3	2.6	4.2	6.7	14.9	2.3	4.2	4.6	8.8	15.8	1.2	2.5	4.2	6.6	14.9
Poorer	1.8	3.3	6.1	10.4	16.6	1.6	3.5	6.8	11.8	19.3	1.8	3.3	6.1	10.2	16.2
Middle	3.0	4.6	7.4	10.9	16.0	3.7	5.1	7.8	13.5	18.6	2.9	4.5	7.3	10.0	15.1
Richer	5.7	7.8	12.6	12.5	17.6	6.3	8.8	13.6	13.4	20.3	5.4	7.3	11.7	11.7	15.4
Richest	15.3	17.3	21.8	16.9	22.1	16.5	18.5	23.1	17.8	23.8	12.1	14.1	17.1	13.7	18.2
**Demand satisfied**															
Poorest	55.4	52.6	52.6	59.5	66.7	58.0	61.5	51.2	60.5	65.9	55.3	52.4	52.6	59.5	66.8
Poorer	58.0	62.5	67.5	71.9	73.1	59.6	64.4	64.7	69.9	69.9	57.9	62.4	67.8	72.2	73.5
Middle	61.0	69.0	74.6	74.8	75.9	63.3	66.7	72.8	71.9	73.6	60.6	69.4	75.0	75.8	76.8
Richer	66.6	72.0	76.7	75.5	77.1	67.0	70.6	76.6	73.5	75.3	66.5	72.7	76.8	77.5	78.6
Richest	70.4	73.2	77.8	76.3	77.6	70.6	73.1	77.7	75.9	77.4	69.9	73.7	78.2	77.7	78.3

**Table 2.** Trends in coverage of FP indicators among currently married women aged 15–49 years by completed years of schooling across place of residence in India, 1992–2021.

	Total					Urban					Rural				
	1992–93	1998–99	2005–06	2015–16	2019–21	1992–93	1998–99	2005–06	2015–16	2019–21	1992–93	1998–99	2005–06	2015–16	2019–21
**mCPR**															
No education	31.3	38.4	45.7	49.0	60.2	38.2	46.7	55.3	54.9	61.4	30.1	37.0	43.6	47.6	59.9
1–10 years	44.5	48.3	51.2	49.7	58.0	49.6	54.3	57.0	53.6	61.1	41.4	45.2	47.9	47.6	56.6
11–12 years	43.1	44.5	48.7	41.4	49.7	47.3	49.2	52.6	46.8	55.4	36.1	38.1	43.8	36.7	45.9
12+ years	46.7	48.4	51.7	40.5	48.2	48.6	50.3	54.2	44.6	53.2	37.8	41.5	44.2	32.8	41.6
**MNRM**															
No education	29.3	35.8	40.8	43.0	48.9	34.4	41.6	46.9	45.9	48.3	28.4	34.8	39.5	42.3	49.0
1–10 years	36.2	39.3	39.6	37.3	39.8	38.4	42.0	42.3	40.0	41.7	34.8	37.8	38.0	36.0	39.0
11–12 years	24.2	26.9	26.9	25.0	25.9	26.0	28.9	27.6	28.7	30.6	21.2	24.3	25.8	21.7	22.8
12+ years	16.9	18.5	19.6	19.2	19.5	16.9	18.8	19.3	21.3	22.0	16.7	17.7	20.4	15.2	16.1
**MRM**															
No education	2.1	3.0	4.9	5.9	10.6	3.8	5.4	8.4	9.0	12.6	1.7	2.6	4.1	5.2	10.2
1–10 years	8.3	9.3	11.7	12.3	17.4	11.2	12.5	14.8	13.6	18.9	6.5	7.6	9.9	11.5	16.7
11–12 years	18.9	17.8	21.9	16.3	22.8	21.3	20.7	25.0	17.9	24.2	15.0	13.9	18.0	14.9	21.9
12+ years	29.8	30.3	32.3	21.2	27.9	31.7	32.0	35.1	23.3	30.5	21.2	24.1	23.9	17.4	24.4
**Demand satisfied**															
No education	60.9	65.2	69.5	75.4	77.7	67.2	71.1	77.2	79.1	77.9	59.6	64.2	67.7	74.4	77.7
1–10 years	66.9	69.3	71.2	72.9	75.2	71.1	73.5	76.6	75.9	77.1	64.2	67.0	68.0	71.2	74.3
11–12 years	61.1	61.1	67.2	64.0	67.9	63.6	66.3	71.9	68.7	72.5	56.3	53.7	61.0	59.5	64.6
12+ years	62.9	65.6	70.3	63.1	67.1	64.6	67.3	72.3	67.1	71.5	54.3	58.8	63.8	54.9	60.7

### Inequality in modern contraceptive use by household wealth and education level

Household wealth-based inequality in all the selected FP outcomes has decreased over time, both within urban and rural areas (Table 3). For instance, the value of the concentration index for MRM decreased from 0.664 in 1992–93 to 0.236 in 2019–21. Similarly, for mCPR, the gap between richest to poorest quintiles decreased from 23% to 11%, and the ratio of richest to poorest decreased from 1.83 to 1.22 between 1992–93 to 2019–21. Though wealth-based inequality decreased over time, inequality persists in all FP outcomes, within both urban and rural areas. Moreover, the inequality was slightly higher in urban than in rural areas. For instance, in 2019–21 the concentration index for mCPR was 0.127 overall, 0.158 in urban areas and 0.143 in rural areas. Similarly, the concentration index for MNRM was 0.153 overall, 0.205 in urban areas and 0.190 in rural areas. The trend in inequality by women’s educational status showed a similar pattern (Table 4). However, except for MRM, education-based inequality in mCPR, MNRM, and demand satisfied was in favour of uneducated/less-educated women. For instance, in 2019–21, the concentration index for MNRM was −0.076 overall, −0.099 in urban areas, and −0.063 in rural areas. Similarly, the gap in mCPR between most- to least-educated women was −12% overall, −8% in urban areas and −18% in rural areas. The ratio of most- to least-educated women was in a similar direction.

**Table 3.** Trends in wealth quintile-based inequity in FP indicators among currently married women aged 15–49 years across place of residence in India, 1992–2021.

	Total					Urban					Rural				
	1992–93	1998–99	2005–06	2015–16	2019–21	1992–93	1998–99	2005–06	2015–16	2019–21	1992–93	1998–99	2005–06	2015–16	2019–21
**mCPR**															
Richest–poorest gap (%)	22.6	27.0	26.8	19.4	11.0	18.7	17.9	26.4	17.3	12.0	21.7	27.1	26.9	20.2	10.1
Richest/poorest ratio	1.83	1.96	1.84	1.56	1.22	1.59	1.48	1.82	1.47	1.25	1.80	1.97	1.84	1.59	1.21
Concentration index	0.257	0.240	0.196	0.182	0.127	0.311	0.263	0.217	0.202	0.158	0.247	0.253	0.218	0.211	0.143
**MNRM**															
Richest–poorest gap (%)	8.6	12.0	9.2	9.3	4.7	4.4	3.4	8.1	8.4	5.7	10.8	15.1	14.0	13.1	7.8
Richest/poorest ratio	1.33	1.46	1.33	1.33	1.14	1.15	1.10	1.29	1.30	1.19	1.42	1.58	1.50	1.47	1.24
Concentration index	0.196	0.192	0.157	0.168	0.153	0.302	0.266	0.228	0.217	0.205	0.216	0.230	0.202	0.214	0.190
**MRM**															
Richest–poorest gap (%)	14.1	14.8	17.7	10.2	7.2	14.2	14.3	18.5	9.0	8.0	10.9	11.6	12.9	7.1	3.3
Richest/poorest ratio	11.98	6.74	5.25	2.53	1.48	7.32	4.39	5.00	2.02	1.50	9.82	5.57	4.11	2.08	1.22
Concentration index	0.664	0.574	0.472	0.281	0.236	0.677	0.613	0.496	0.378	0.312	0.489	0.500	0.432	0.348	0.222
**Demand satisfied**															
Richest–poorest gap (%)	15.0	20.7	25.3	16.8	10.9	12.7	11.5	26.6	15.3	11.4	14.6	21.4	25.6	18.2	11.5
Richest/poorest ratio	1.27	1.39	1.48	1.28	1.16	1.22	1.19	1.52	1.25	1.17	1.26	1.41	1.49	1.31	1.17
Concentration index	0.127	0.124	0.115	0.101	0.081	0.175	0.154	0.127	0.114	0.099	0.126	0.136	0.132	0.119	0.094

**Table 4.** Trends in education-based (years of schooling) inequity in FP indicators among currently married women aged 15–49 years across place of residence in India, 1992–2021.

	Total					Urban					Rural				
	1992–93	1998–99	2005–06	2015–16	2019–21	1992–93	1998–99	2005–06	2015–16	2019–21	1992–93	1998–99	2005–06	2015–16	2019–21
**mCPR**															
12+ years–no education gap (%)	15.4	10.0	6.1	−8.5	−12.0	10.4	3.6	−1.1	−10.3	−8.2	7.7	4.4	0.6	−14.8	−18.3
12+ years/no education ratio	1.49	1.26	1.13	0.83	0.80	1.19	1.10	1.01	0.93	0.95	1.26	1.12	1.01	0.69	0.69
Concentration index	0.276	0.197	0.105	0.056	0.013	0.123	0.066	0.040	0.017	0.010	0.346	0.254	0.151	0.070	0.014
**MNRM**															
12+ years–no education gap (%)	−12.4	−17.3	−21.2	−23.8	−29.5	−17.4	−22.8	−27.6	−24.6	−26.4	−11.7	−17.2	−19.2	−27.1	−32.9
12+ years/no education ratio	0.58	0.52	0.48	0.45	0.40	0.98	0.91	0.83	0.78	0.76	0.59	0.51	0.52	0.36	0.33
Concentration index	0.218	0.135	0.018	−0.020	−0.076	0.005	−0.048	−0.083	−0.079	−0.099	0.325	0.229	0.107	0.011	−0.063
**MRM**															
12+ years–no education gap (%)	27.8	27.3	27.5	15.3	17.3	27.8	26.6	26.7	14.3	18.0	19.4	21.5	19.8	12.2	14.2
12+ years/no education ratio	14.54	10.07	6.66	3.58	2.64	3.05	2.52	2.01	1.69	1.70	12.23	9.20	5.82	3.35	2.40
Concentration index	0.606	0.528	0.428	0.331	0.264	0.439	0.389	0.344	0.254	0.225	0.633	0.487	0.456	0.349	0.282
**Demand satisfied**															
12+ years–no education gap (%)	2.0	0.3	0.8	−12.2	−10.6	−2.6	−3.8	−4.9	−12.0	−6.4	−5.4	−5.3	−3.8	−19.5	−16.9
12+ years/no education ratio	1.03	1.01	1.01	0.84	0.86	1.02	1.00	0.98	0.93	0.97	0.91	0.92	0.94	0.74	0.78
Concentration index	0.101	0.081	0.043	0.010	0.002	0.032	0.018	0.012	0.114	0.005	0.158	0.116	0.064	0.015	0.002

### Multivariate analysis

Results of multivariate analysis showed that the predicted probability of modern contraceptive use and demand satisfied is significantly associated with household wealth quintiles (Table 5) and level of education among women (Table 6) even after adjusting for the pertinent covariates. For instance, in 2019–21, the predicted probability of mCPR was 0.484 among the poorest women, 0.536 among poorer women, 0.571 among middle women, 0.581 among richer women, and 0.583 among the richest women. The pattern of association remained similar in the previous rounds of the survey and within urban and rural areas. Regarding women’s education, we observed an inverse association with mCPR, MNRM, and demand satisfied—the higher the level of education, the lower the predicted probability of these outcomes. However, in the case of MRM, the association was direct—the predicted probability of MRM was 0.109 among uneducated women, 0.181 among women with 1–10 years of schooling, 0.239 among women with 11–12 years of schooling, and 0.285 among women with 12+ years of schooling in 2019–21. This pattern was consistent across the survey rounds and urban–rural residence.

**Table 5.** Predicted probabilities^a^ [95% confidence interval] showing effect of household wealth quintiles on FP indicators among currently married women aged 15–49 years across place of residence in India, 1992–2021.

	Total					Urban					Rural				
	1992–93	1998–99	2005–06	2015–16	2019–21	1992–93	1998–99	2005–06	2015–16	2019–21	1992–93	1998–99	2005–06	2015–16	2019–21
**mCPR**															
Poorest	0.265	0.275	0.325	0.348	0.484	0.294	0.373	0.314	0.358	0.481	0.264	0.272	0.326	0.348	0.484
	[0.258, 0.272]	[0.267, 0.282]	[0.317, 0.333]	[0.345, 0.351]	[0.481, 0.487]	[0.261, 0.326]	[0.328, 0.417]	[0.276, 0.353]	[0.344, 0.372]	[0.465, 0.497]	[0.257, 0.271]	[0.265, 0.279]	[0.318, 0.334]	[0.345, 0.350]	[0.481, 0.487]
Poorer	0.292	0.347	0.424	0.431	0.536	0.321	0.362	0.402	0.423	0.517	0.290	0.346	0.427	0.432	0.538
	[0.285, 0.299]	[0.339, 0.354]	[0.416, 0.431]	[0.429, 0.434]	[0.534, 0.539]	[0.296, 0.346]	[0.332, 0.392]	[0.381, 0.423]	[0.415, 0.423]	[0.507, 0.526]	[0.282, 0.297]	[0.338, 0.353]	[0.419, 0.435]	[0.429, 0.435]	[0.535, 0.541]
Middle	0.335	0.429	0.498	0.472	0.571	0.355	0.422	0.465	0.442	0.542	0.332	0.430	0.511	0.482	0.578
	[0.329, 0.342]	[0.422, 0.436]	[0.491, 0.505]	[0.469, 0.475]	[0.569, 0.574]	[0.337, 0.373]	[0.404, 0.440]	[0.452, 0.478]	[0.436, 0.448]	[0.535, 0.540]	[0.325, 0.339]	[0.422, 0.437]	[0.503, 0.519]	[0.479, 0.485]	[0.575, 0.582]
Richer	0.409	0.490	0.546	0.488	0.581	0.417	0.491	0.540	0.466	0.569	0.406	0.489	0.554	0.507	0.588
	[0.403, 0.416]	[0.483, 0.497]	[0.539, 0.552]	[0.485, 0.491]	[0.578, 0.584]	[0.406, 0.429]	[0.480, 0.502]	[0.531, 0.548]	[0.461, 0.470]	[0.564, 0.574]	[0.399, 0.414]	[0.481, 0.497]	[0.544, 0.564]	[0.502, 0.511]	[0.584, 0.592]
Richest	0.502	0.549	0.580	0.520	0.583	0.513	0.554	0.586	0.517	0.585	0.471	0.533	0.549	0.526	0.580
	[0.496, 0.508]	(0.542, 0.555]	[0.570, 0.586]	[0.516, 0.523]	[0.580, 0.586]	[0.506, 0.520]	[0.546, 0.561]	[0.580, 0.593]	[0.513, 0.521]	[0.581, 0.589]	[0.459, 0.483]	[0.520, 0.545]	[0.534, 0.564]	[0.520, 0.533]	[0.574, 0.585]
**MNRM**															
Poorest	0.252	0.256	0.284	0.282	0.323	0.273	0.340	0.270	0.276	0.303	0.251	0.254	0.285	0.282	0.323
	[0.245, 0.258]	[0.249, 0.263]	[0.277, 0.292]	[0.279, 0.284]	[0.320, 0.325]	[0.242, 0.304]	[0.297, 0.383]	[0.234, 0.306]	[0.263, 0.289]	[0.289, 0.317]	[0.244, 0.258]	[0.247, 0.261]	[0.277, 0.293]	[0.279, 0.284]	[0.321, 0.326]
Poorer	0.273	0.317	0.355	0.330	0.360	0.295	0.321	0.317	0.307	0.315	0.271	0.317	0.360	0.332	0.364
	[0.266, 0.279]	[0.310, 0.324]	[0.348, 0.362]	[0.327, 0.332]	[0.357, 0.363]	[0.272, 0.319]	[0.293, 0.350]	[0.297, 0.337]	[0.299, 0.315]	[0.306, 0.323]	[0.263, 0.278]	[0.309, 0.324]	[0.353, 0.368]	[0.330, 0.335]	[0.361, 0.347]
Middle	0.300	0.381	0.402	0.353	0.396	0.305	0.363	0.361	0.308	0.343	0.300	0.384	0.417	0.367	0.408
	[0.294, 0.307]	[0.374, 0.387]	[0.395, 0.409]	[0.350, 0.356]	[0.393, 0.399]	[0.288, 0.322]	[0.346, 0.380]	[0.348, 0.373]	[0.303, 0.314]	[0.337, 0.349]	[0.293, 0.306]	[0.376, 0.391]	[0.410, 0.425]	[0.364, 0.370]	[0.405, 0.411]
Richer	0.343	0.402	0.404	0.343	0.387	0.341	0.396	0.396	0.316	0.352	0.344	0.406	0.415	0.367	0.407
	[0.337, 0.349]	[0.396, 0.409]	[0.397, 0.410]	[0.340, 0.346]	[0.384, 0.390]	[0.330, 0.352]	[0.385, 0.407]	[0.388, 0.404]	[0.312, 0.320]	[0.347, 0.357]	[0.337, 0.351]	[0.398, 0.414]	[0.405, 0.424]	[0.363, 0.371]	[0.403, 0.411]
Richest	0.321	0.358	0.348	0.323	0.339	0.316	0.350	0.347	0.313	0.325	0.336	0.384	0.352	0.351	0.362
	[0.315, 0.327]	[0.352, 0.365]	[0.342, 0.353]	[0.320, 0.326]	[0.336, 0.342]	[0.310, 0.323]	[0.343, 0.357]	[0.340, 0.353]	[0.309, 0.316]	[0.322, 0.329]	[0.324, 0.347]	[0.372, 0.396]	[0.338, 0.366]	[0.345, 0.356]	[0.357, 0.367]
**MRM**															
Poorest	0.014	0.024	0.041	0.065	0.146	0.020	0.044	0.044	0.079	0.159	0.013	0.023	0.041	0.065	0.145
	[0.012, 0.015]	[0.021, 0.027]	[0.038, 0.045]	[0.064, 0.067]	[0.144, 0.148]	[0.009, 0.031]	[0.023, 0.065]	[0.026, 0.063]	[0.070, 0.088]	[0.147, 0.172]	[0.011, 0.015]	[0.021, 0.026]	[0.037, 0.045]	[0.063, 0.066]	[0.143, 0.147]
Poorer	0.019	0.033	0.069	0.101	0.165	0.026	0.043	0.085	0.115	0.191	0.019	0.033	0.067	0.099	0.163
	[0.017, 0.022]	[0.031, 0.036]	[0.065, 0.073]	[0.099, 0.102]	[0.163, 0.168]	[0.016, 0.035]	[0.029, 0.057]	[0.072, 0.098]	[0.109, 0.121]	[0.183, 0.199]	[0.017, 0.021]	[0.030, 0.036]	[0.062, 0.071]	[0.097, 0.101]	[0.161, 0.165]
Middle	0.035	0.052	0.097	0.119	0.168	0.050	0.064	0.104	0.133	0.193	0.033	0.049	0.094	0.114	0.162
	[0.032, 0.038]	[0.048, 0.055]	[0.092, 0.101]	[0.116, 0.121]	[0.166, 0.170]	[0.041, 0.059]	[0.054, 0.074]	[0.096, 0.113]	[0.129, 0.137]	[0.187, 0.198]	[0.030, 0.036]	[0.046, 0.053]	[0.089, 0.099]	[0.112, 0.116]	[0.160, 0.165]
Richer	0.066	0.090	0.142	0.144	0.188	0.076	0.097	0.144	0.149	0.211	0.062	0.086	0.140	0.140	0.175
	[0.063, 0.070]	[0.086, 0.094]	[0.137, 0.147]	[0.141, 0.146]	[0.186, 0.191]	[0.069, 0.083]	[0.090, 0.105]	[0.138, 0.150]	[0.145, 0.152]	[0.206, 0.215]	[0.058, 0.066]	[0.081, 0.091]	[0.132, 0.147]	[0.136, 0.143]	[0.172, 0.178]
Richest	0.181	0.193	0.234	0.196	0.239	0.197	0.207	0.241	0.204	0.255	0.135	0.151	0.197	0.175	0.212
	[0.176, 0.186]	[0.187, 0.198]	[0.228, 0.239]	[0.193, 0.199]	[0.236, 0.242]	[0.191, 0.203]	[0.200, 0.213]	[0.235, 0.247]	[0.201, 0.207]	[0.251, 0.259]	[0.126, 0.144]	[0.141, 0.161]	[0.185, 0.210]	[0.170, 0.180]	[0.208, 0.217]
**Demand satisfied**															
Poorest	0.542	0.521	0.537	0.596	0.661	0.557	0.615	0.505	0.594	0.649	0.541	0.517	0.538	0.596	0.661
	[0.531, 0.553]	[0.510, 0.532]	[0.526, 0.548]	[0.592, 0.599]	[0.658, 0.664]	[0.506, 0.607]	[0.556, 0.675]	[0.451, 0.559]	[0.574, 0.613]	[0.631, 0.668]	[0.530, 0.553]	[0.506, 0.529]	[0.527, 0.549]	[0.592, 0.600]	(0.658, 0.664)
Poorer	0.576	0.615	0.652	0.679	0.716	0.606	0.627	0.601	0.663	0.680	0.573	0.614	0.660	0.681	0.719
	[0.565, 0.587]	[0.605, 0.625]	[0.643, 0.662]	[0.676, 0.682]	[0.713, 0.719]	[0.569, 0.643]	[0.586, 0.667]	[0.574, 0.627]	[0.652, 0.673]	[0.669, 0.690]	[0.562, 0.584]	[0.604, 0.625]	[0.650, 0.669]	[0.677, 0.684]	(0.716, 0.722)
Middle	0.609	0.668	0.712	0.711	0.747	0.623	0.654	0.666	0.673	0.711	0.607	0.670	0.730	0.723	0.756
	[0.600, 0.618]	[0.660, 0.676]	[0.705, 0.720]	[0.708, 0.714]	[0.744, 0.750]	[0.598, 0.648]	[0.632, 0.676]	[0.651, 0.682]	[0.666, 0.680]	[0.732, 0.743]	[0.597, 0.617]	[0.661, 0.679]	[0.721, 0.738]	[0.719, 0.727]	(0.753, 0.759)
Richer	0.662	0.699	0.746	0.722	0.757	0.663	0.694	0.739	0.698	0.737	0.662	0.702	0.756	0.742	0.769
	[0.654, 0.670]	[0.691, 0.706]	[0.739, 0.753]	[0.719, 0.726]	[0.754, 0.760]	[0.649, 0.678]	[0.682, 0.707]	[0.730, 0.748]	[0.693, 0.704]	[0.732, 0.743]	[0.653, 0.671]	[0.692, 0.711]	[0.745, 0.766]	[0.737, 0.746]	(0.765, 0.773)
Richest	0.693	0.722	0.777	0.742	0.755	0.698	0.723	0.781	0.738	0.751	0.677	0.719	0.756	0.752	0.762
	[0.686, 0.700]	[0.715, 0.729]	[0.771, 0.783]	[0.739, 0.746]	[0.752, 0.759]	[0.690, 0.706]	[0.715, 0.731]	[0.774, 0.788]	[0.734, 0.742]	[0.747, 0.755]	[0.663, 0.690]	[0.706, 0.732]	[0.740, 0.771]	[0.746, 0.759]	(0.757, 0.768)

Predicted probabilities are obtained after running multivariate analysis adjusted for age and education of women, number of living children, caste, religion, exposure to FP message through media, and region of the country.

**Table 6.** Predicted probabilities^a^ [95% of confidence intervals] showing effect of completed years of schooling on FP indicators among currently married women aged 15–49 years across place of residence in India, 1992–2021.

	Total					Urban					Rural				
	1992–93	1998–99	2005–06	2015–16	2019–21	1992–93	1998–99	2005–06	2015–16	2019–21	1992–93	1998–99	2005–06	2015–16	2019–21
**mCPR**															
No education	0.323	0.391	0.471	0.464	0.594	0.392	0.476	0.540	0.510	0.606	0.308	0.375	0.446	0.455	0.592
	[0.319, 0.327]	[0.387, 0.396]	[0.466, 0.476]	[0.462, 0.466]	[0.592, 0.596]	[0.383, 0.402]	[0.465, 0.487]	[0.530, 0.549]	[0.504, 0.515]	[0.599, 0.612]	[0.304, 0.313]	[0.370, 0.380]	[0.440, 0.452]	[0.453, 0.458]	[0.590, 0.595]
1–10 years	0.443	0.477	0.513	0.454	0.555	0.499	0.545	0.558	0.491	0.584	0.404	0.434	0.473	0.439	0.547
	[0.438, 0.448]	[0.472, 0.482]	[0.509, 0.518]	[0.452, 0.456]	[0.553, 0.557]	[0.491, 0.507]	[0.537, 0.553]	[0.551, 0.564]	[0.487, 0.495]	[0.580, 0.588]	[0.398, 0.411]	[0.428, 0.440]	[0.467, 0.479]	[0.436, 0.441]	[0.545, 0.549]
11–12 years	0.441	0.445	0.481	0.389	0.480	0.480	0.497	0.513	0.439	0.536	0.366	0.356	0.420	0.354	0.453
	[0.425, 0.458]	[0.429, 0.460]	[0.468, 0.493]	[0.384, 0.393]	[0.475, 0.484]	[0.459, 0.501]	[0.478, 0.517]	[0.498, 0.529]	[0.432, 0.446]	[0.528, 0.543]	[0.339, 0.393]	[0.332, 0.380]	[0.399, 0.440]	[0.349, 0.360]	[0.448, 0.458]
12+ years	0.485	0.480	0.516	0.383	0.465	0.509	0.501	0.535	0.430	0.516	0.365	0.387	0.431	0.320	0.417
	[0.470, 0.501]	[0.465, 0.494]	[0.505, 0.527]	[0.379, 0.388]	[0.461, 0.469]	[0.492, 0.526]	[0.485, 0.517]	[0.523, 0.547]	[0.424, 0.436]	[0.510, 0.522]	[0.328, 0.401]	[0.354, 0.419]	[0.405, 0.456]	[0.313, 0.326]	[0.412, 0.423]
**MNRM**															
No education	0.295	0.359	0.411	0.399	0.477	0.332	0.416	0.448	0.408	0.467	0.287	0.348	0.397	0.398	0.479
	[0.291, 0.299]	[0.355, 0.363]	[0.406, 0.416]	[0.397, 0.402]	[0.475, 0.480]	[0.323, 0.341]	[0.405, 0.426]	[0.438, 0.457]	[0.403, 0.414]	[0.460, 0.474]	[0.283, 0.291]	[0.344, 0.353]	[0.392, 0.403]	[0.395, 0.400]	[0.476, 0.481]
1–10 years	0.344	0.371	0.377	0.324	0.365	0.366	0.403	0.401	0.342	0.378	0.328	0.351	0.355	0.317	0.361
	[0.339, 0.349]	[0.366, 0.376]	[0.373, 0.381]	[0.322, 0.326]	[0.363, 0.366]	[0.359, 0.374]	[0.395, 0.410]	[0.394, 0.407]	[0.339, 0.345]	[0.374, 0.382]	[0.322, 0.335]	[0.345, 0.357]	[0.349, 0.361]	[0.315, 0.319]	[0.359, 0.363]
11–12 years	0.220	0.246	0.239	0.210	0.229	0.231	0.268	0.251	0.243	0.271	0.200	0.209	0.217	0.187	0.210
	[0.206, 0.234]	[0.232, 0.259]	[0.228, 0.250]	[0.206, 0.213]	[0.226, 0.233]	[0.213, 0.248]	[0.250, 0.285]	[0.237, 0.264]	[0.237, 0.249]	[0.265, 0.278]	[0.177, 0.223]	[0.188, 0.230]	[0.200, 0.234]	[0.183, 0.192]	[0.206, 0.214]
12+ years	0.153	0.167	0.182	0.154	0.169	0.154	0.170	0.184	0.176	0.193	0.148	0.154	0.170	0.124	0.147
	[0.142, 0.165]	[0.156, 0.178]	[0.173, 0.190]	[0.151, 0.157]	[0.166, 0.172]	[0.142, 0.167]	[0.157, 0.182]	[0.175, 0.194]	[0.171, 0.180]	[0.188, 0.197]	[0.120, 0.175]	[0.129, 0.178]	[0.150, 0.190]	[0.120, 0.129]	[0.143, 0.151]
MRM															
No education	0.028	0.036	0.060	0.064	0.109	0.060	0.064	0.092	0.101	0.133	0.021	0.031	0.048	0.057	0.106
	[0.026, 0.029]	[0.035, 0.038]	[0.058, 0.063]	[0.063, 0.065]	[0.108, 0.111]	[0.005, 0.065]	[0.058, 0.070]	[0.086, 0.098]	[0.097, 0.104]	[0.128, 0.138]	[0.020, 0022]	[0.029, 0.033]	[0.046, 0.051]	[0.056, 0.058]	[0.104, 0.107]
1–10 years	0.099	0.109	0.137	0.129	0.181	0.132	0.144	0.158	0.148	0.200	0.076	0.086	0.118	0.121	0.176
	[0.096, 0.102]	[0.106, 0.112]	[0.133, 0.140]	[0.127, 0.130]	[0.180, 0.183]	[0.126, 0.138]	[0.138, 0.150]	[0.152, 0.163]	[0.146, 0.151]	[0.197, 0.203]	[0.072, 0.080]	[0.082, 0.090]	[0.113, 0.122]	[0.119, 0.122]	[0.174, 0.178]
11–12 years	0.221	0.202	0.242	0.178	0.239	0.249	0.233	0.263	0.196	0.258	0.166	0.151	0.203	0.166	0.230
	[0.206, 0.236]	[0.189, 0.215]	[0.231, 0.254]	[0.174, 0.181]	[0.235, 0.242]	[0.230, 0.268]	[0.215, 0.250]	[0.249, 0.278]	[0.190, 0.202]	[0.251, 0.264]	[0.143, 0.189]	[0.132, 0.170]	[0.186, 0.221]	[0.161, 0.170]	[0.225, 0.234]
12+ years	0.332	0.317	0.337	0.229	0.285	0.354	0.335	0.353	0.254	0.316	0.217	0.237	0.262	0.194	0.257
	[0.317, 0.347]	[0.303, 0.331]	[0.326, 0.348]	[0.225, 0.233]	[0.281, 0.289]	[0.338, 0.371]	[0.319, 0.351]	[0.341, 0.365]	[0.248, 0.259]	[0.310, 0.321]	[0.184, 0.249]	[0.207, 0.266]	[0.238, 0.285]	[0.189, 0.200]	[0.252, 0.262]
**Demand satisfied**															
No education	0.615	0.656	0.710	0.730	0.772	0.665	0.706	0.757	0.754	0.772	0.603	0.645	0.691	0.725	0.772
	[0.609, 0.621]	[0.650, 0.661]	[0.705, 0.716]	[0.727, 0.732]	[0.769, 0.774]	[0.653, 0.677]	[0.693, 0.718]	[0.747, 0.767]	[0.748, 0.760]	[0.766, 0.779]	[0.596, 0.609]	[0.639, 0.651]	[0.684, 0.698]	[0.722, 0.728]	[0.769, 0.774]
1–10 years	0.661	0.680	0.715	0.688	0.728	0.700	0.725	0.755	0.720	0.745	0.630	0.648	0.676	0.675	0.722
	[0.655, 0.667]	[0.675, 0.686]	[0.710, 0.719]	[0.686, 0.691]	[0.726, 0.730]	[0.691, 0.709]	[0.716, 0.733]	[0.748, 0.762]	[0.716, 0.724]	[0.741, 0.749]	[0.622, 0.638]	[0.641, 0.655]	[0.669, 0.683]	[0.672, 0.678]	[0.720, 0.725]
11–12 years	0.617	0.614	0.667	0.609	0.660	0.643	0.665	0.702	0.661	0.707	0.559	0.521	0.598	0.571	0.637
	[0.598, 0.636]	[0.596, 0.632]	[0.653, 0.681]	[0.604, 0.615]	[0.656, 0.665]	[0.620, 0.667]	[0.643, 0.686]	[0.685, 0.719]	[0.653, 0.670]	[0.699, 0.715]	[0.524, 0.593]	[0.491, 0.551]	[0.573, 0.622]	[0.564, 0.578]	[0.632, 0.643]
12+ years	0.640	0.647	0.705	0.600	0.649	0.659	0.665	0.729	0.646	0.692	0.534	0.558	0.628	0.530	0.606
	[0.623, 0.658]	[0.631, 0.663]	[0.693, 0.717]	[0.594, 0.605]	[0.644, 0.653]	[0.640, 0.677]	[0.647, 0.683]	[0.707, 0.733]	[0.639, 0.653]	[0.686, 0.699]	[0.488, 0.579]	[0.518, 0.598]	[0.598, 0.658]	[0.522, 0.539]	[0.599, 0.612]

^a^Predicted probabilities are obtained after running multivariate analysis adjusted for age and education of women, number of living children, caste, religion, exposure to FP message through media, and region of the country.

**Figure 2.**
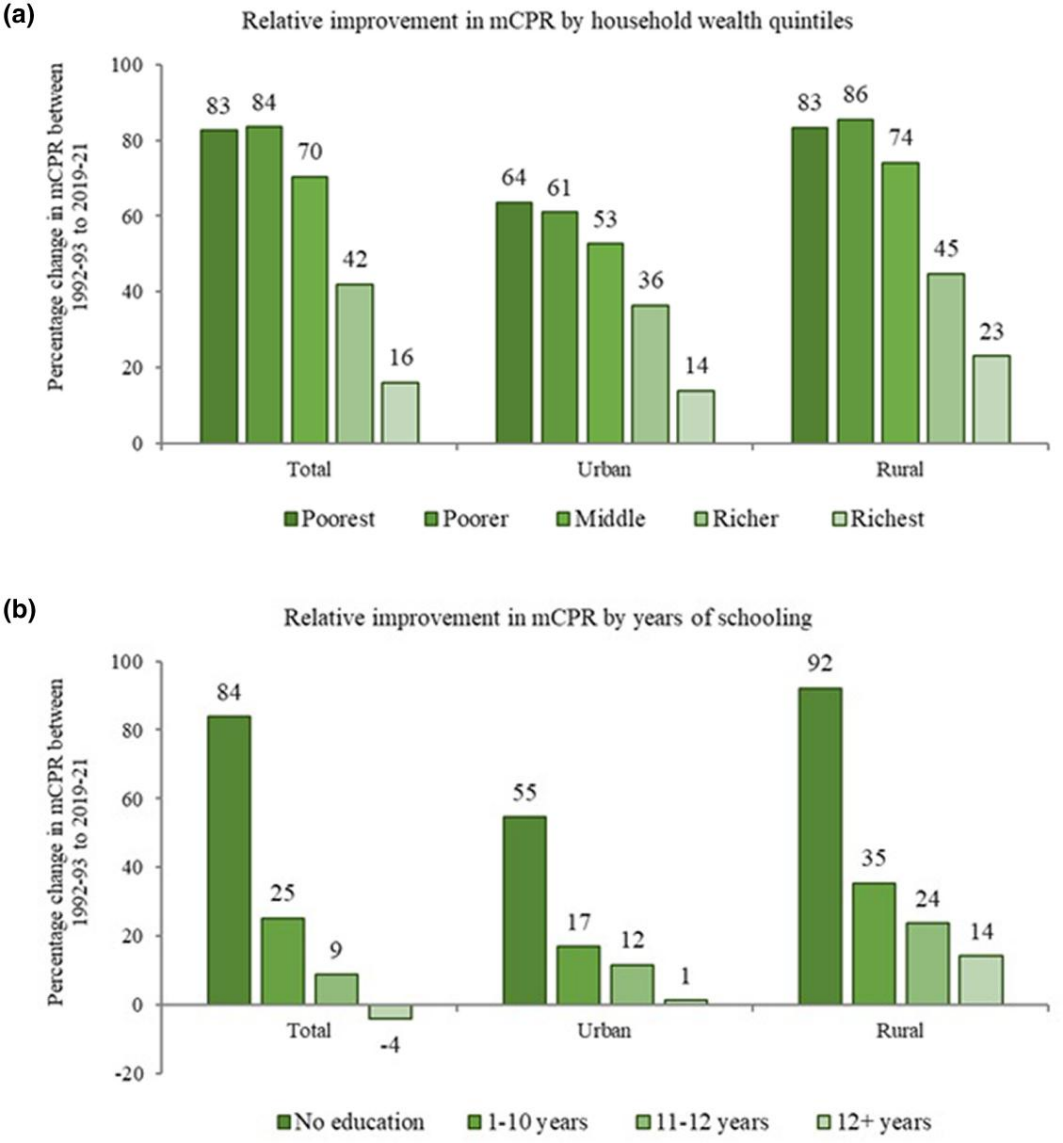
Percentage change in probability of mCPR among currently married women aged 15–49 years by (**a**) household wealth quintiles and (**b**) years of schooling, across place of residence in India, 1992–2021. Predicted probabilities were obtained after running multivariate analysis adjusted for age and education of women, number of living children, caste, religion, exposure to FP message through media, and region of the country.

Relative changes in the predicted probability between 1992–93 to 2019–21 showed that the increase in mCPR was higher among women from the poorest compared with the richest quintile—the increase was 83% among women from the poorest quintile, 84% among the poorer quintile, 70% among the middle quintile, 42% among the richer quintile, and 16% among the richest quintile (Fig. 2a). This pattern was similar within urban and rural areas. For instance, within urban areas, the increase was 64% among women of the poorest quintile and 14% among the richest quintile, and within rural areas, the increase was 83% among the poorest and 23% in the richest quintile. During the same period, the predicted probability of mCPR increased 84% among women with no education, 25% among women educated for 1–10 years, 9% among women educated for 11–12 years, but declined −4% among women with 12+ years of schooling (Fig. 2b). This pattern was similar for other outcomes as well (Appendix 1).

## Discussion

In India, modern contraceptive use and demand satisfied has increased over time and currently more than half of married women are using modern contraceptives. Modern contraceptive use has also increased within urban and rural areas. Notably, the urban–rural gap, initially favouring urban women, has minimized over time. These improvements in FP indicators in the country might be a result of India’s long journey of FP and well-being programmes till the recent commitment of FP2020 and FP2030, which focused on the marginalized population.

The substantial improvement in FP indicators across household wealth quintiles and education level, within both urban and rural areas, suggests that the increase in modern contraceptive use over time is the result of FP programmes that benefit women of all wealth quintiles and of all education levels. Furthermore, the increase was greater among poor and less-educated compared with rich and more-educated women, respectively. This has resulted in a decline in overall inequality in modern contraceptive use and demand satisfied in the country, within both urban and rural areas. Many previous studies have highlighted a narrowing rich–poor gap in contraceptive use across developing countries (Creanga et al. 2011, Fotso et al. 2013, Mutua et al. 2021). This narrowing inequality in modern contraceptive use is a reflection of the success of FP programmes and highlights that disadvantaged populations may have benefited the most from FP programmes offering increased contraceptive access and a range of choices. This finding also likely reflects a change in the propensity to implement fertility desires through uptake of modern methods in the country.

The relative improvement in the use of modern methods was higher among marginalized women in rural areas than among their urban counterparts. This is likely because the FP programme in India also focuses on demand generation, specifically within rural areas through extensive engagement of frontline health workers, such as Accredited Social Health Activists (ASHAs). These frontline health workers make door-to-door visits to inform and counsel women, particularly those who are poor and marginalized, on FP methods, and escort those women to nearby public health facilities to adopt clinical methods (Kumar et al. 2020a). However, there are no such dedicated health workers for demand generation within urban areas. Hence, focused demand-generation within rural areas might have resulted in a higher relative change in modern contraceptive adoption among marginalized rural women.

Despite the increase in contraceptive use and demand satisfied among women across the wealth and education groups, socioeconomic inequality still exists. This persistent inequality in contraceptive prevalence is also noticed in many Asian, African, and low- and middle-income countries (Gakidou and Vayena 2007, Gillespie et al. 2007, Aslam et al. 2016, Ponce de Leon et al. 2019, Mutua et al. 2021, Sreeramareddy et al. 2022). Both wealth- and education-based inequality was greater in the use of modern reversible contraceptives than other methods, indicating that wealthier and educated women rely more on the use of reversible methods, which they mostly obtained from the private sector, compared with their poorer and less-educated counterparts (Fotso et al. 2013, Kumar et al. 2020b). It is well argued that private-sector interventions may increase inequality in contraceptive use as the cost of FP services charged to the clients in the private sector may influence economic access, with high prices discouraging poor women from using such services (Agha and Do 2008). On the other hand, the greater reliance of poor women on female sterilization may be because women chose an irreversible alternative to avoid limitations in access, the inconvenience of frequent use of a reversible method, and the lack of side-effects of irreversible methods, to some extent. Wealth-related inequality in contraceptive use was slightly higher among urban compared with rural women. This may be because of the higher number of private sector outlets in urban compared with rural areas, that are largely accessible to women of the highest wealth group; so increased use of contraceptives in urban areas may expand overall inequality unless it is counter-balanced by increased contraceptive use in rural areas (Agha and Do 2008, Karen et al. 2010).

Inequality by women’s education showed a different pattern—pro-poor inequality in the use of MNRMs, minimal inequality in the use of any modern method and demand satisfied, and persistent pro-rich inequality in the use of MRMs. This pattern holds true within urban and rural areas. This could be because most educated women are better informed about the range of choices available in the health system, and their sources, and may have better financial access to the reversible methods (pills, condoms, intrauterine contraceptive devices, and injectables). Furthermore, educated women are more likely to obtain services from a private facility, which is likely to be associated with higher inequality (Kumar et al. 2020b). On the other hand, less-educated women are likely to use female sterilization in India, which is mostly obtained from public health facilities (Blumenberg et al. 2020, Singh et al. 2021). This may be the result of a lack of comprehensive knowledge about the choices and sources that exist. Furthermore, high reliance on female sterilization might be a result of a favourable policy environment, the influence of health providers, and reinforced supply- and demand-side incentives related to this method (Edmeades et al. 2011, Ugaz et al. 2016).

Our analyses have some limitations, notably the findings are based on cross-sectional data, which may not allow the demonstration of a causal relationship between programme efforts and an increase in contraceptive use among poor and uneducated women. Second, for the purposes of this analysis we have grouped all types of reversible methods (intrauterine contraceptive device, condom, pills, injectables etc.) together due to the issue of sample size required to analyse the data for a specific method; hence one might expect differing patterns of economic inequality between an intrauterine contraceptive device versus pills/condoms. However, given that pills/condoms contribute >90% to the total MRMs, we assume that this may not affect our results. Third, household wealth indices might vary according to the choice of assets, which is affected by issues of comparability between urban and rural households. Fourth, comparison of women’s education across time may be subject to question, as 1–10 years of schooling in 1992–93 may have different implications for a woman’s life compared to achieving the same level of education during 2019–21. Fifth, in the regression analysis, we did not include certain covariates such as women’s occupation, decision-making, mobility etc. because these indicators are only available for a subsample of the women, hence including these variables could significantly reduce the size of the analytical sample.

## Conclusion

The consistent increase in contraceptive use in India and among women of low socioeconomic status is indeed good news and reflects the success of long-standing FP programmes in the country which are increasingly reaching the marginalized population. This has resulted in minimizing the gap between poor and rich, and between the less- and most-educated women. Despite this success, current levels of contraceptive use among the poorest women are similar to the level that the richest women reached two decades before. This indicates that the ongoing FP programme in the country should increasingly target marginalized sections of the population to bring them to the level where their counterparts stand now. This will help India achieve the universal access to reproductive health services required to achieve Sustainable Development Goal 3.7.

The persistent inequality, particularly for MRMs, underscores the need to increase the availability and accessibility of MRMs among the marginalized population, along with improving the information given to them and the method-mix offered. This will help in balancing the method-mix in the country, which has for decades been dominated by female sterilization. In any case, it is the woman who should decide which method to opt for, given a free and informed choice on contraception. The higher inequality in modern contraceptive use within urban compared with rural areas, though slight, requires attention in the context of a rapidly growing urban population, particularly as our findings showed better improvement in adoption of modern methods among the marginalized population of rural areas, which may be the result of the focused programmatic effort for demand-generation activities within rural areas. A similar dedicated programmatic effort for demand generation within urban communities may help poor and marginalized urban women to adopt modern methods to a greater extent and hence contribute to decreasing inequality within urban areas.

## Data Availability

The data underlying this article will be shared on reasonable request to the corresponding author.

## Reflexivity statement

The authors include one female and at least one male and span multiple levels of seniority. While four of the authors specialize in family planning research in India another is a scientist with expertise in family planning and reproductive health across the globe. All five authors have extensive experience in conducting quantitative research in India.

## Appendix

**Appendix 1.** Percentage change (relative change) in probability^a^ of FP indicators among currently married women aged 15–49 years by household quintiles and completed years of schooling across place of residence in India, 1992–2021

	mCPR			MNRM			MRM			Demand satisfied		
	Total	Urban	Rural	Total	Urban	Rural	Total	Urban	Rural	Total	Urban	Rural
**Household wealth quintiles**												
Poorest	82.6	63.6	83.3	28.2	11.0	28.7	942.9	695.0	1015.4	22.0	16.5	22.2
Poorer	83.6	61.1	85.5	31.9	6.8	34.3	768.4	634.6	757.9	24.3	12.2	25.5
Middle	70.4	52.7	74.1	32.0	12.5	36.0	380.0	286.0	390.9	22.7	14.1	24.5
Richer	42.1	36.5	44.8	12.8	3.2	18.3	184.8	177.6	182.3	14.4	11.2	16.2
Richest	16.1	14.0	23.1	5.6	2.8	7.7	32.0	29.4	57.0	8.9	7.6	12.6
**Women years of schooling**												
No education	83.9	54.6	92.2	61.7	40.7	66.9	289.3	121.7	404.8	25.5	16.1	28.0
1–10	25.3	17.0	35.4	6.1	3.3	10.1	82.8	51.5	131.6	10.1	6.4	14.6
11–12	8.8	11.7	23.8	4.1	17.3	5.0	8.1	3.6	38.6	7.0	10.0	14.0
12+	–4.1	1.4	14.2	10.5	25.3	–0.7	–14.2	–10.7	18.4	1.4	5.0	13.5

^a^Predicted probabilities were obtained after running multivariate analysis adjusted for age and education of women, number of living children, caste, religion, exposure to FP messages through media, and region of the country.
